# Machine learning to improve interpretability of clinical, radiological and panel-based genomic data of glioma grade 4 patients undergoing surgical resection

**DOI:** 10.1186/s12967-023-04308-y

**Published:** 2023-07-07

**Authors:** Michele Dal Bo, Maurizio Polano, Tamara Ius, Federica Di Cintio, Alessia Mondello, Ivana Manini, Enrico Pegolo, Daniela Cesselli, Carla Di Loreto, Miran Skrap, Giuseppe Toffoli

**Affiliations:** 1 Experimental and Clinical Pharmacology Unit, Centro di Riferimento Oncologico di Aviano (CRO) IRCCS, 33081 Aviano, Italy; 2 Neurosurgery Unit, Head-Neck and Neuroscience Department, University Hospital of Udine, 33100 Udine, Italy; 3 Institute of Pathology, University Hospital of Udine, 33100 Udine, Italy; 4 Department of Medicine, University of Udine, 33100 Udine, Italy

**Keywords:** Glioma, Machine learning, Prognosis, Carmustine wafer, Tumor mutational burden

## Abstract

**Background:**

Glioma grade 4 (GG4) tumors, including astrocytoma IDH-mutant grade 4 and the astrocytoma IDH wt are the most common and aggressive primary tumors of the central nervous system. Surgery followed by Stupp protocol still remains the first-line treatment in GG4 tumors. Although Stupp combination can prolong survival, prognosis of treated adult patients with GG4 still remains unfavorable. The introduction of innovative multi-parametric prognostic models may allow refinement of prognosis of these patients. Here, Machine Learning (ML) was applied to investigate the contribution in predicting overall survival (OS) of different available data (e.g. clinical data, radiological data, or panel-based sequencing data such as presence of somatic mutations and amplification) in a mono-institutional GG4 cohort.

**Methods:**

By next-generation sequencing, using a panel of 523 genes, we performed analysis of copy number variations and of types and distribution of nonsynonymous mutations in 102 cases including 39 carmustine wafer (CW) treated cases. We also calculated tumor mutational burden (TMB). ML was applied using eXtreme Gradient Boosting for survival (XGBoost-Surv) to integrate clinical and radiological information with genomic data.

**Results:**

By ML modeling (concordance (c)- index = 0.682 for the best model), the role of predicting OS of radiological parameters including extent of resection, preoperative volume and residual volume was confirmed. An association between CW application and longer OS was also showed. Regarding gene mutations, a role in predicting OS was defined for mutations of *BRAF* and of other genes involved in the PI3K-AKT-mTOR signaling pathway. Moreover, an association between high TMB and shorter OS was suggested. Consistently, when a cutoff of 1.7 mutations/megabase was applied, cases with higher TMB showed significantly shorter OS than cases with lower TMB.

**Conclusions:**

The contribution of tumor volumetric data, somatic gene mutations and TBM in predicting OS of GG4 patients was defined by ML modeling.

**Supplementary Information:**

The online version contains supplementary material available at 10.1186/s12967-023-04308-y.

## Introduction

Glioma grade 4 (GG4) tumors, which include astrocytoma IDH-mutant grade 4 and the astrocytoma IDH wt (that currently defines the glioblastoma class) are the most common and aggressive primary tumors of the central nervous system [1–3]. Compelling evidence, based on objective tumor volume analysis, supports the role of the extent of resection (EOR) in high grade glioma patients as the first step of patients management. Surgical treatment, however, can rarely be considered as radical, due to infiltrating nature, multifocal presentation, and ill-defined tumor margins [3–5]. Although the Stupp protocol was introduced as postoperative standard treatment more than 15 years ago, alternative treatments have not been recently developed, and the poor 5-year survival rate has not changed significantly in these last decades [6–9]. The infiltrative growing, the rapid proliferative rate of malignant cells and the appearance of treatment-resistant cell clones shortly after initial therapy induces recurrence within 2 cm of resection margins, regardless the initial EOR. In addition GG4 are characterized by high spatial and temporal molecular heterogeneity, which implies a poor prognosis, especially in glioblastoma cases with a median survival of less than 15 months [1, 2, 6–8, 10–12].

The intraoperative treatment with Carmustine Wafers (CW) implantation [marketed as Gliadel, biodegradable copolymers discs impregnated with the alkylating agent Bis-ChloroethylNitrosoUrea (BCNU)], for newly high grade glioma was introduced in 2003 as a therapeutic bridge between surgery and Stupp protocol onset. After an initial enthusiasm, its employment has been gradually reduced, mainly for contrasting results in terms of efficacy and safety, but not completely abandoned [13–19].

The differences in overall survival (OS) and response to treatment are largely due to the wide heterogeneity of GG4, with a variable distribution of biological features associated with aggressiveness between tumors as well as within a single tumor. Several prognostic factors for GG4 have been proposed including *MGMT* promoter methylation status, *IDH1*, *IDH2* mutation, EOR and residual volume [10, 20–23]. In addition, comprehensive multiplatform genome-wide analyses have demonstrated that GG4 heterogeneity is dependent from specific molecular/genetic features [12, 24–26]. Finally, a high tumor mutation burden (TMB) in glioma has been significantly associated with short OS [27]. However, the prognostic value of TBM in GG4 has not been fully elucidated [27–29].

In the last years, machine learning (ML) techniques have been applied in the oncology field with the aim of improving diagnosis, prediction and prognosis of cancers, being characterized by the capability of taking in account interaction effects and nonlinearities. In particular, several ML techniques have been adapted in order to be applied on censored data for survival analyses. In this context, survival tree methods are non-parametric, flexible methods that have been developed with the aim of dealing with high dimensional covariate data [30–32].

In the present study, with the aim of better defining prognostic markers for OS, we performed a comprehensive molecular characterization of surgical specimens of a monoistitutional cohort of 102 patients who underwent surgical resection of newly GG4 and adjuvant postoperative Stupp protocol. By a next-generation sequencing (NGS) approach using a broad exome panel of 523 genes, analysis of copy number variations and of types and distribution of nonsynonymous mutations was performed. ML models were developed by using eXtreme Gradient Boosting for survival (XGBoost-Surv) to investigate the contribution in predicting OS of the different available data (e.g. clinical data including follow-up, radiological data, or molecular data derived by panel-based sequencing such as presence of somatic mutations and amplification).

## Methods

### Patient cohort

The study included 102 patients who underwent a surgical resection of a newly diagnosed GG4 at the Neurosurgery Department of Udine Hospital between 2014 and 2019 [1–3]. Median age of diagnosis was 60 years. Written informed consent was obtained for surgery. Patients provided informed consent in accordance with the local institutional review board requirements and the Declaration of Helsinki. After surgery, all patients were treated with combinations of concomitant adjuvant radiotherapy and chemotherapy, followed by adjuvant chemotherapy, as recommended by Stupp [7, 8], and were included in a study approved by the local Ethics Committee (protocol N. 0036566 /P/ GEN/EGAS, ID study 2538). Treatment with CW was applied in 39 out of 102 GG4 cases. All the 102 cases were characterized for the main clinical, radiological and molecular parameters with prognostic significance that were reported in Additional file 1: Table S1 [20–23].

### DNA extraction

DNA material was extracted from frozen GG4 sample sections using the Allprep DNA / RNA / miRNA Universal Kit (Qiagen). Alternatively, DNA was extracted from formalin-fixed, paraffin-embedded (FFPE) GG4 samples using the AllPrep DNA / RNA FFPE Kit (Qiagen).

### Sequencing analysis and “Tumor Mutational Burden” (TMB) determination

The 102 GG4 cases included in the series were all sequenced using the TruSight Oncology 500 DNA kit (TSO500). The library was prepared manually according to the manufacturer’s protocol. NGS was performed using the NextSeq 550 instrument (Illumina) with eight libraries per sequencing run. Further information regarding sequencing analysis and determination of TMB is reported in the Additional file 1: Methods section S1.

The calculation of the “Tumor Mutational Burden” (TMB) using the data obtained from sequencing employing the TSO500 panel (Illumina) was determined by using the local TruSight Oncology 500 version 2.3 (Illumina) app. The manufacturer’s quality control criteria were used to determine whether a TMB determination result with TSO500 was valid, including NGS library concentration ≥ 1 ng / µL, median insert size ≥ 70 bp, median coverage exon ≥ 50, count and percentage of exons with coverage of at least 50 counts ≥ 90%. TMB metrics were calculated following small variant calling. TMB was determined as the ratio of the number of eligible variants (mutations) to eligible DNA regions [expressed in megabases (Mb)]. Eligible variants (representing the numerator) include only coding variants with a frequency ≥ 5% and coverage ≥ 50 reads. Single Nucleotide Variants (SNV) and insertion/deletion (indel) were included, but multi-nucleotide variants (MNVs) and variants with a cosmic count ≥ 50 were excluded. Variants in blacklisted regions with poor mapability were also excluded. For the denominator, all eligible coding regions (with coverage ≥ 50x) were included, except for the blacklist regions. Small variants were exported from the TSO500 pipeline. All passed filter data, including TMB and “small variants” (SNV and indel) were provided in the CombinedVariantOutput.tsv file for each sample. All the small variants calls were merged into one file. An additional quality control for the call of “small variants”, TMB, copy number variations (CNV) was used to select samples with the best quality.

### Evaluation of the TMB in the TCGA cohort

The tumor mutational burden (TMB) was calculated from the MC3 Public MAF file as previously described [33]. Only primary tumors from the high grade glioma TCGA cohort were included in the analysis [25].

### Machine learning approach for survival prediction

The approach used to predict patient OS was based on the application of ML methods [31, 32, 34]. The datasets were composed by integrating different information. The main resource variables, divided into clinical (radiological and clinical variables), and molecular including gene mutation status, and copy number results (amplifications and deletions), are reported in Additional file 1: Table S2.

EXtreme Gradient Boosting for survival (XGBoost-Surv) modeling t was performed in Python (V3.8) using the xgboost and sklearn_surv library partially evaluated using the scikit-learn library [35]. In each of the datasets, string-based categorical variables were converted to numerical values, and each continuous variable was standardized. All XGBoost models were trained using the survival Cox objective function. Additionally, for each dataset, hyperparameter tuning was performed using the HyperOpt package [36, 37]. The mean Harrell’s concordance index (c-index) was computed using the five-fold cross-validation (CV) approach and used as metric [31, 38, 39]. After identifying the hyperparameters, the model was subsequently evaluated on the same total dataset using five-fold CV with the randomly selected folds being distinct from those used in the tuning of the hyperparameters to accurately assess the generalizability of the model [31]. Further details have been included as Additional Information.

Because of the high number of variables in the datasets used compared with the number of cases, the dimensionality of the input in the datasets was reduced by feature selection. In this context, selection was applied firstly considering all mutations included in the output and then used the merged TMB trace to filter out germline variants. Other variants were excluded by filtering the mutation type. Specifically, only in the case in which a mutation found by the sequencing was considered to perturb the gene (i.e., missense variant, stop loss, start loss), the mutation and therefore the gene were selected. Thus, only genes with at least one non-silent mutation were recorded into a presence or absence mutation (binary-valued patients)-by-genes matrix. Multiple hit mutations and singular mutations were both indicated as presence of mutation (representing in both cases a mutated status for the gene). Of note, from this list of variants that was generated, clinical data were integrated into the previous produced matrix.

In detail, to perform ML modeling, 4 different datasets were created. Two datasets (dataset 1 and dataset 2) were created considering the whole series of the 102 GG4 cases (Additional file 1: Tables S3 and S4). Two other datasets (dataset 3 and 4) were created in the context of a subgroup of 71 out of 102 cases that were characterized by values above the 75 percentile for the following parameters: median insert size, median coverage exon, and PCT_exon 50x (Additional file 1: Tables S3 and Table S4). Specifically, in dataset 1 all the available variables arisen from the 102 cases were considered. In dataset 2 and dataset 3, feature selection was applied by selecting somatic gene mutations present in at least 4 GG4 cases. For both dataset 2 and dataset 3, the selection of most important features was obtained by using the EXtreme Gradient Boosting for survival (XGBoost-Surv) modeling t performed in Python (V3.8), which provides a way to compute feature importance by measuring how concordance index (c-index) decreases when a feature is not available. In the survival framework, the remotion of the relationship of a certain feature with the survival time is executed by random shuffling of its values: the weight of each feature is quantified by the drop on average of the c-index [40]. Finally, dataset 4 was obtained by applying expert selection, by considering only genes included in the PI3K-AKT-mTOR signaling pathway (WP3844) [41].

In order to understand how the models yielded their predictions, Shapley Additive exPlanations (SHAP) values were used to obtain a visualization of the overall feature importance for the models [30–32]. Then, SHAP dependence plots were generated for each model in order to compare how the features contributed to the corresponding model’s output.

## Results

### Impact of established prognosticators in the whole GG4 cohort

The main clinical and molecular markers with established prognostic significance in the 102 GG4 cases entering the study cohort are reported in Additional file 1: Table S1. Median age of diagnosis was 60 years. Eight/102 (7.8%) GG4 cases were *IDH* mutated and 94/102 cases (92.2%) were *IDH* wild type. Sixty-two/102 GG4 cases (60.8%) showed a methylated *MGMT* promoter whereas 40/102 GG4 cases showed an unmethylated *MGMT* promoter. Median OS was 15.5 months (range 2–48 months). Treatment with CW was applied during surgical resection in 39/102 GG4 cases (38.2%).

By univariate Cox regression analysis, preoperative volume (hazard ratio (HR) = 1.10, p = 0.033), EOR (HR = 0.92, p < 0.001), a methylated status of the *MGMT* promoter (HR = 0.44, p < 0.001) and CW treatment (HR = 0.53, p = 0.003) showed a significant impact on OS. In a multivariate Cox regression analysis model in which all parameters with significant impact in the univariate Cox regression analysis were included, EOR (HR = 0.85, p = 0.002), *MGMT* promoter methylation status (HR = 0.47, p < 0.001) and CW treatment (HR = 0.62, p = 0.032) retained an impact on OS (Table 1) [10, 13–20].

**Table 1:** Univariate and multivariate Cox regression analysis of OS in the whole cohort (102 GG4 cases)

Characteristic	N	Univariate			Multivariate		
		HR	95% CI	p-value	HR	95% CI	p-value
CW	102						
0; untreated	63	–	–		–	–	
1; treated	39	0.53	0.34, 0.81	**0.003**	0.62	0.40, 0.96	**0.032**
Age	102	1.01	1.0, 1.04	0.2			
EOR	102	0.92	0.89, 0.96	**< 0.001**	0.94	0.91, 0.98	**0.002**
Localization	102						
0; precentral	41	–	–				
1; postcentral	21	1	0.58, 1.72	> 0.9			
2; temporoinsular	40	1.09	0.70, 1.71	0.7			
Ki67	102	1.01	1.00, 1.02	0.14			
*IDH* mutational status	102						
0; unmutated	94	–	–				
1; mutated	8	0.65	0.32, 1.35	0.3			
Side	102						
0; left	54	–	–				
1; right	48	1.2	0.81, 1.80	0.4			
Preoperative volume	102	1.01	1.00, 1.02	**0.033**	1.01	1.00, 1.02	0.25
Extent of resection_2 categories	102						
0; ≤99%	52	–	–				
1; ≥100%	50	0.81	0.54, 1.20	0.3			
*MGMT* status	102						
0; unmethylated	40	–	–				
1; methylated	62	0.44	0.28, 0.68	**< 0.001**	0.47	0.30,0.74	**< 0.001**
Gender: female	102	1.18	0.76, 1.82	0.5			

*CI* confidence interval, *HR* hazard ratio, *EOR* extent of resection. In bold p-value < 0.05

### Impact of molecular and clinical variables on OS by a ML approach

**Fig. 1:**
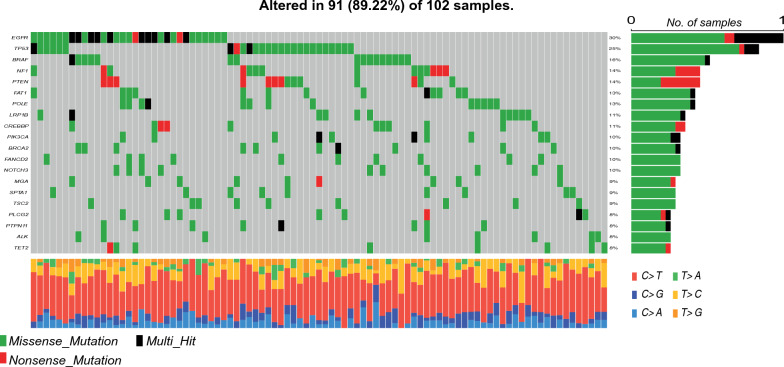
Distribution and classification of the gene mutations found in the 102 sequenced GG4 cases. The distribution of the type of mutations (e.g. missense mutations, nonsense mutations and multihit) and mutation frequencies for the 20 genes most frequently found mutated are shown. The number of mutated cases for each gene is also shown

The most frequently found gene mutations for the 102 cases are shown in Fig. 1. In particular, the 3 most frequently altered genes were *EGFR*, *TP53* and *BRAF* [25]. The gene with the higher rate of amplification was *EGFR* gene (Additional file 1: Figure S1) [25].

To account for genomic features in predicting OS of GBM cases, we used a ML approach to build a multiparametric clinical-molecular prediction model that includes clinical information, somatic mutations, and amplifications [31, 32, 34, 42]. In this ML modeling, functional mutations and amplifications were considered only on the basis of their presence or absence, regardless of their specific location, according to the generated simple matrix, as reported in methods’ section.

**Fig. 2:**
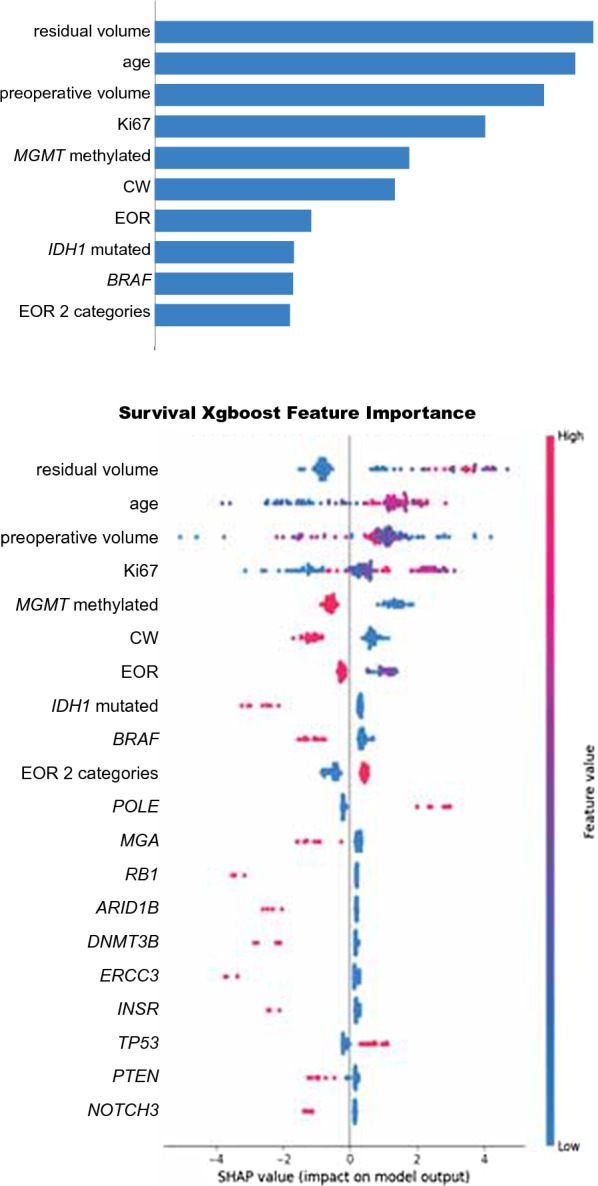
Feature importance ranked by “mean absolute magnitude” of SHAP values using dataset 2. The model was developed for the 102 GG4 series using 107 variables constituted by 95 genes and 12 clinical/radiological variables (dataset 2). Upper panel: mean absolute values corresponding to the magnitude of feature importance. Lower panel: summary plots for SHAP values; for each considered feature, a single patient is represented by one point. Along the x axis the position of a point corresponds to the logarithm of the mortality risk associated with that feature for a specific patients. This value corresponds to the impact that the feature had on the model output for that specific patient. Data clusters with SHAP values around zero indicate low impact on the model. Along the y axis, the different features are disposed according to their importance corresponding to the mean of their absolute SHAP values. Features with the higher importance are disposed on the upper part of the summary plots. SHAP, Shapley Additive exPlanation

To perform ML modeling, several feature selection methods were employed. By using this approach, we obtained 2 datasets (dataset 1 and dataset 2) for the entire cohort of 102 GG4 cases and 2 datasets (dataset 3 and dataset 4) for the subset of 71 patients in which TMB calculation met the appropriate metrics according to the Illumina pipeline (Additional file 1: Tables S3 and S4). A first preliminary comprehensive model developed using 417 gene mutations, 45 gene amplifications and 12 clinical/radiological variables (dataset 1) showed a concordance - index (c-index) of 0.537 in the test set. To better define the contribute of the most relevant somatic mutations in predicting OS, in the context of a model that also considered clinical variables, the number of gene mutations was reduced by selecting those present in at least 4 GG4 cases. By this approach, the model developed using 107 variables constituted by 95 gene mutations and 12 clinical/radiological variables (dataset 2) showed the best c-index (c-index of 0.682 in the test set, Additional file 1: Table S3). Figure 2 shows the SHAP analysis reporting the first 20 variables that explain the model developed using dataset 2. The directions in the SHAP plots of these variables showed a significant contribution in predicting OS for the well-established prognosticators: residual volume, preoperative volume, EOR and methylation status of *MGMT*, also in keeping with results of Cox regression analysis. With this model, *IDH1* mutation status had also a prognostic significance [10, 20, 23, 43]. The direction in the SHAP plots was also consistent with the multivariate Cox regression analysis for the treatment with CW, that was considered as a protective variable [13–19]. SHAP analysis in Fig. 2 also reported the contribution of gene mutations in predicting OS. Among gene mutations, the higher contribution was reported for *BRAF*. Several other genes such as *POLE*, *PTEN*, *NOTCH3* and *TP53* emerged as important for OS prediction [24–26, 44–49].

**Fig. 3:**
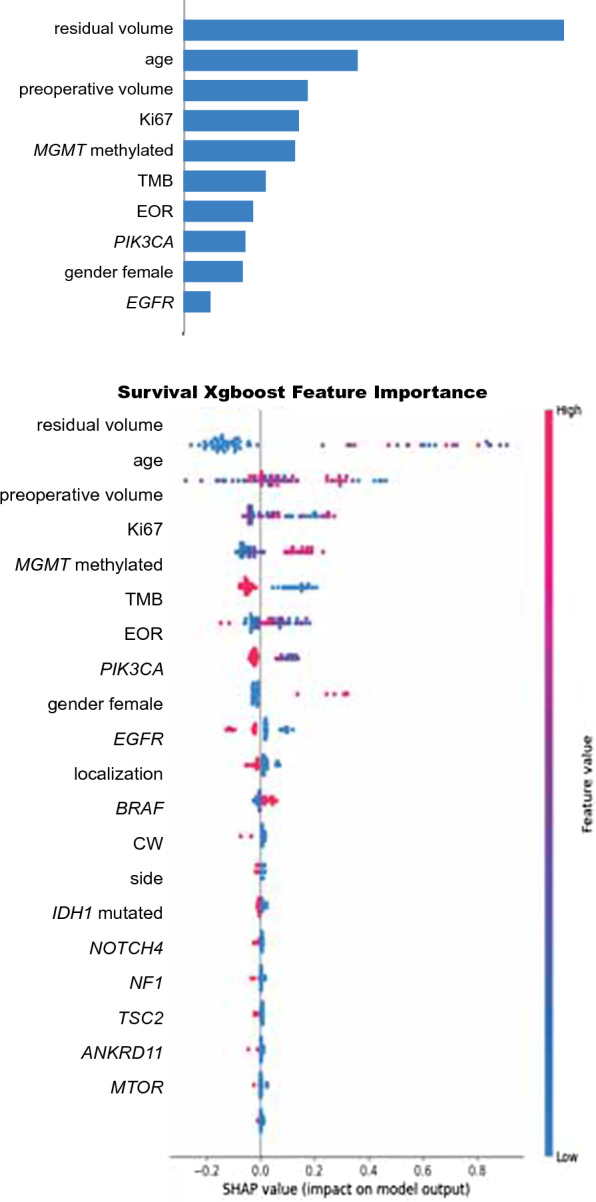
Feature importance ranked by “mean absolute magnitude” of SHAP values using dataset 3. The model was developed for the 71 GG4 cases with available TMB values using 108 variables constituted by 95 genes, 12 clinical/radiological variables and TMB (dataset 3). Upper panel: mean absolute values corresponding to the magnitude of feature importance. Lower panel: summary plots for SHAP values; for each considered feature, a single patient is represented by one point. Along the x axis the position of a point corresponds to the logarithm of the mortality risk associated with that feature for a specific patients. This value corresponds to the impact that the feature had on the model output for that specific patient. Data clusters with SHAP values around zero indicate low impact on the model. Along the y axis, the different features are disposed according to their importance corresponding to the mean of their absolute SHAP values. Features with the higher importance are disposed on the upper part of the summary plots. SHAP, Shapley Additive exPlanation

To evaluate the effect not only of genes but also of the new emerging biomarker TMB as OS prognostic indicator, a further dataset (dataset 3) was considered (Additional file 1: Tables S3 and S4). In detail, by using dataset 3, composed by 108 features constituted by 95 gene mutations, 12 clinical/radiological variables, and TMB (dataset 3), a model with a c-index of 0.625 in the test set was obtained (Additional file 1: Table S3). Of note, SHAP analysis highlighted the importance for the predicting of OS for TMB, with higher TMB values associated with shorter OS (Fig. 3).

**Fig. 4:**
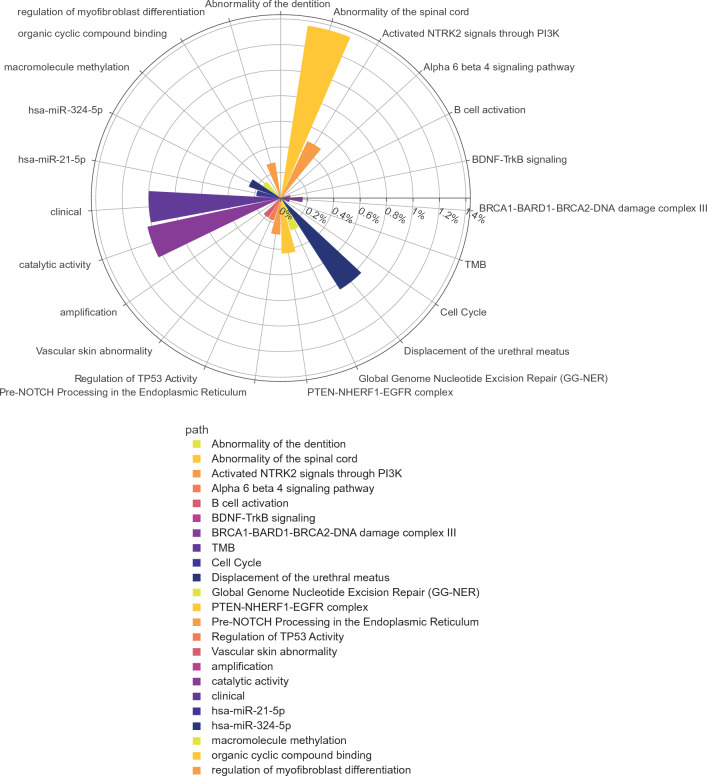
Contribution of clinical, radiological, and molecular data in predicting survival of GG4 cases by ML. Circulating barplot, obtained by overall considering the results of the 3 different ML models (obtained with dataset 1, dataset 2 and dataset 3), recapitulating the overall contribution in explaining GG4 clinical outcome of clinical/radiological parameters, TMB values and dysregulated signaling pathways by detected gene mutations

The results of the 3 ML models (dataset1, dataset 2, dataset 3, Additional file 1: Tables S3, S4) were summarized by a circular barplot to comprehensively consider variable importance of clinical and radiological variables, TMB and signaling pathways putatively dysregulated by gene mutations. Figure 4 shows a main contribution for clinical and radiological variables together with a contribution for genes belonging to several categories including “activated NTRK2 signals through PI3K”. These results confirmed the importance of clinical and radiological variables in predicting OS of GG4 cases as well as a role for TMB. Moreover, these results confirmed the role of gene mutations involved in the PI3K pathway alteration in affecting the clinical outcome of GG4 cases [25, 50].

Recently, several signaling pathways have been highlighted as a potential focus for therapeutic intervention strategies [51]. Among these, a key role was attributed to the PI3K-AKT-mTOR signaling pathway [50]. Thus, to further investigate the role of the PI3K-AKT-mTOR pathway, we restricted the ML approach to the genes belonging to this pathway (WP3844)[41], in a dataset also comprising clinical and radiological data, and TMB data (dataset 4; Additional file 1: Tables S3 and S4). In this model, 27 features were included, constituted by 16 genes, 10 clinical and surgical variables and TMB (dataset 4). With this approach, a c-index of 0.670 in the test set was obtained (Additional file 1: Table S3). SHAP analysis confirmed the importance of clinical and radiological variables and further suggested a role for TMB in predicting OS (Additional file 1: Figure 2A, B). Moreover, SHAP analysis defined a contribution of genes belonging to PI3K-AKT-mTOR signaling pathway in particular *BRAF* and *PI3KCA* (Additional file 1: Figure S2A) [25, 44, 50].

### Impact of TMB as OS prognosticator

**Fig. 5:**
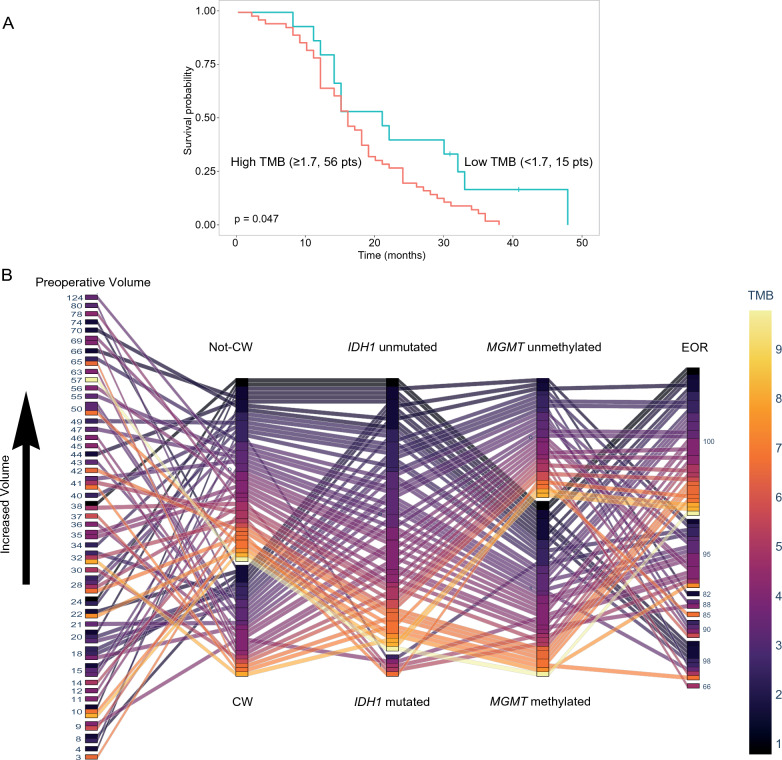
Impact of TMB in the clinical outcome of GG4 cases. **A** Impact of TMB on survival in the series of 71 GG4 cases for which TMB calculation met the appropriate metrics according to the Illumina pipeline. Kaplan-Meier curves comparing the OS intervals of GG4 cases with high TMB (TMB ≥ 1.7 mutations/MB, 56 cases, red line) and GG4 cases with low TMB (TMB < 1.7 mutations/MB, 15 cases, blu line). The p-value reported refers to the log-rank test. **B** Line graph recapitulating the association between clinical/radiological parameters and TMB values for each GG4 case

Based on the results of the ML modeling, the role of TMB as OS prognostic indicator was further explored to define a TMB cutoff to separate GG4 cases into 2 subgroups with different OS. In the context of the 71 GG4 cases in which the TMB calculation met the appropriate metrics, maximally selected log-rank statistics identified the cutoff value of 1.7 mutations/MB as the most appropriate cutoff value (not shown). By dividing this 71 GG4 cases into 2 categories, 56 cases were included in the TMB category ≥ 1.7 mutations/MB and 15 cases were included in the TMB category < 1.7 mutations/MB. By using this cutoff, GG4 cases with a TMB ≥ 1.7 mutations/MB (median OS = 16 months) had shorter OS compared with GG4 cases with a TMB < 1.7 mutations/MB (median OS = 21 months, p = 0.047, Fig. 5A). Of note, in our GG4 series, high TMB values were frequently present in GG4 cases characterized by other biomarker of worse prognosis (Fig. 5B). Finally, using the cutoff value of 1.7 mutations/MB for TMB, a difference (although not significant) was also observed in the context of high grade glioma cases from the TCGA series in which, however, data were obtained by a whole exome sequencing approach at difference from the panel-based sequencing approach we used (Additional file 1: Figure S3).

## Discussion

Newly diagnosed GG4 represents a heterogeneous group of brain tumors, characterized by minimally effective genotype-targeted therapies, in which surgery followed by Stupp protocol is still the current first-line treatment despite decades of research [1, 2, 6–8]. In the present study, a comprehensive molecular characterization using a NGS approach with a wide panel of 523 genes was performed in the context of a mono-institutional cohort of newly diagnosed GG4 patients who underwent neurosurgery followed by Stupp protocol and CW treatment. Then, ML modeling was applied with the aim of defining the prognostic value of clinical, radiological and molecular parameters in predicting OS.

Several challenges need to be overcome in order to define prognostic markers for OS in cancer diseases, including GG4. They mainly include the different variables to be considered and the way these variables interact with each other. This has became crucial with the introduction of NGS approaches capable to generate high amount of genomic data of which interpretation could become complex [30–32]. To overcome this complexity, ML approaches have been proposed in cancer research. In our study a ML approach was applied using XGBoost to develop multi-parametric predictor models for survival analysis [31, 32, 34]. Moreover, to describe the contribution of each variable to prediction, SHAP values were employed [30–32].

We chose Cox regression analysis as the starting point for our study to confirm the prognostic significance of established prognosticators for survival in the context of our GG4 case cohort, as this analysis is the most commonly used approach for survival analysis in a variety of fields, including oncology, where it is mainly used to identify the prognostic factors that have an impact on patient occurrence or survival [52]. However, Cox regression analysis is generally considered an inappropriate model for high-dimensional associations and relies on some other restrictive assumptions such as proportionality of hazard functions for any two patients and uncorrelated characteristics. Furthermore, Cox regression analysis is not able to model non-linearities and interaction effects [30–32]. In this context, we introduced ML modeling to incorporate the large amount of genomic data obtained by NGS using the panel of 523 genes in the analysis [30–32].

In particular, the ML approach allowed us to take in consideration the specific contribution on the prediction of the high amount of data obtained by the panel-based sequencing regarding the presence of somatic genomic alterations (e.g. somatic mutations and amplifications). Thus, in the present study, different ML models were developed both in the context of the entire cohort of 102 GG4 cases and in the subgroup of 71 GG4 cases in which TMB values were considered useful. Specifically, in addition to the preliminary comprehensive dataset 1 model, the model using dataset 2 focused mainly on determining the contribution of the major somatic mutations as OS prognosticators, when clinical variables were also considered. The dataset 3 model was mainly focused on defining the contribution of TMB as a OS prognosticator. Finally, the dataset 4 model focused specifically on the role of the PI3K-AKT-mTOR pathway given the potential implications for targeted therapeutic strategies.

The intra-operative treatment with CW was developed according to the rational of locally interfering with the potential tumor re-growth in the proximity of the original tumor site [13–19]. Although this treatment option seems to have lost clinical importance in the recent few years, recent long-term follow up investigations have demonstrated a survival benefit in selected cases, shedding thus the light on the effectiveness of this therapeutic option [16]. Despite controversies in current literature [13–19], our data from ML modeling showed that CW treatment was associated with longer OS. This result was also in keeping with multivariate Cox regression analysis. Even if such data prevent to drive final conclusions due to the small sample size, they suggest to further re-consider the application of CW in sequential combination with the Stupp protocol in selected cases to avoid the known side effects (young patients with small lesion and without ventricle opening during surgery) [16].

The ML approach also showed that, for all the developed models, the radiological variables EOR, residual volume and preoperative volume, and the *MGMT* methylated status were located in the top positions of the SHAP diagrams, thus highlighting their role as OS prognosticators in GG4 cases undergoing surgery followed by Stupp protocol, in keeping with previous investigations [10, 20–23].

Considering somatic gene mutations, results of the ML approach showed that a mutated status for different genes was defined as having a role in OS prediction. The outcome of the developed predictor models with different identified genes was dependent by the choices of the employed feature selection. However, in this context, the great majority of altered genes was previously identified as associated with high grade glioma and contributing in the dysregulation of key pathways involving tumor suppressors or oncogenes [25, 44–51, 53]. In particular, a role for gene mutations of *BRAF* was highlighted by the ML modeling. Specifically, the presence of *BRAF* mutations was defined as associated with longer OS, as previously reported [44]. ML modeling also suggested a prognostic relevance for genes involved in the PI3K-AKT-mTOR signaling pathway [50, 53]. This give emphasis for further studies to better clarify the functional impact of the presence of these gene mutations in the progression and treatment resistance of high grade glioma. Moreover, these results could suggest useful targets for personalized therapy for the treatment of patients affected by GG4 [47, 50, 51, 53]. In this context, a panel-driven approach such as that evaluating 523 genes that has been adopted in the present study may allow an accurate molecular diagnosis of tumor specimens of GG4 patients [53, 54]. This panel-driven approach also represents an useful tool for identifying and elucidating complicated pathways such as the PI3K pathway, therefore allowing the repositioning of drugs or the development of novel drugs for personalized therapy. It also allows the possibility of combining therapies targeting different pathways in GG4 [50, 51, 53].

Finally, the application of XGBoost in the subgroup of 71 cases, in which TMB calculation met the appropriate criteria according to the Illumina TMB metrics, suggested an association between a higher TMB and shorter OS [55]. In our study, 1.7 mutations/MB was proposed as the optimal cutoff value for TMB to separate GG4 cases into 2 subgroups with different OS. Different cutoffs have been previously proposed by other studies [27]. The lack of a common cutoff between the different studies might depend on the different intrinsic characteristics of the analyzed series (e.g., volumetric or subjective evaluation of EOR, WHO classification applied, number of needle biopsies included) as well as on the sequencing method (whole exome sequencing, such as in the TCGA cohort, vs. panel-based sequencing). Direct comparisons between TMB cutoffs obtained with different panels can be very problematic, as a TMB cutoff certainly depends on the genomic features and bioinformatics platform used in a given panel [27, 28, 54]. Nevertheless, the correlation between higher TMB and shorter OS found in the present study for GG4 cases is consistent with a previously published analysis of TCGA and CGGA databases [27]. This evidence is in keeping with the concept that a high TMB could be considered as an indicator of a high rate of somatic mutations putatively associated with cell proliferative advantages as well as chemoresistance molecular mechanisms [12, 24–26]. The TMB has been proposed as a predictive marker for response to immunecheckpoint inhibitors [56]. However, an association of high TMB with high responsiveness to immunecheckpoint inhibitor treatment in cohorts of aggressive glioma patients undergoing such immunotherapy remains to be fully elucidated [28, 29, 55–57].

The present study suffers for several limitations mainly due to the retrospective nature of this investigation and the simple size, expecially in the subgroup of 71 GG4 cases with available TMB values., In this context, the retrospective acquisition of data could be associated to the lack of standardized follow-up. However, the fact that the present study analyzed a mono-institutional cohort determined that all the GG4 cases included in this study were homogeneously treated, thus attenuating the possible lack of standardization. On the other hand, the analysis of a mono-institutional cohort could challenge the generalizability of results when an external patient population is considered. The ML approach we employed was chosen in an attempt to overcome these limitations. However, in the present study, our modeling principle was a trade-off between a minimal number of features and the ability to make good predictions, thus avoiding overfitting. Our aim was to strike a balance between interpretability of the model and improved accuracy. Although interpretable models are preferred in the clinical setting, it is possible that a black box model would have resulted in better performance [30–32].

Further integrative analysis, including a MRI radiomic approach may contribute to refine a preoperative prognosis, to plan personalized surgical treatment and to offer patients preoperative counseling. In the future, a prospective multicenter study with a larger sample size is needed to optimize prediction models for clinical practice, and to overcome the intrinsic limitations of retrospective studies.

## Conclusions

In conclusion, the results of the present study showed that the proposed ML approach using XGBoost was capable to define a key role for radiological variables in predicting OS of GG4 cases undergoing surgical resection and Stupp protocol. A protective role for CW treatment was also suggested. Moreover, this approach was capable to recapitulate the contribution as OS prognosticators of somatic mutations of several genes, including *BRAF* and other genes involved in the PI3K-AKT-mTOR signaling pathway. In addition, with the same approach, we showed that, the TMB, as defined by the wide target gene panel of 523 genes, appeared to be associated with shorter OS. Therefore, TMB could be useful to refine the prognosis of GG4 patients undergoing to surgical resection and Stupp protocol.

## Supplementary Information

**Additional file 1: Figure S1.** Distribution and classification of the gene alterations found in the 102 sequenced GG4. cases. A) Distribution of the type of alterations (e.g. missense mutations, nonsense mutations, deletions, amplifications, multihits, complex events) and frequencies for the 20 genes most frequently found altered are shown. The number of sequenced cases found altered for each gene is also shown. **Figure S2.** ML model obtained by considering somatic mutations involving genes belonging to the PI3K-AKT-mTOR signaling pathway (WP3844). A) Feature importance ranked by the "mean absolute magnitude" of the SHAP values of the model obtained for the 71 GG4 cases with available TMB values using a dataset (dataset 4) that included 27 features constituted by 16 genes, 10 clinical and surgical variables and the biomarker TMB. This selection was chosen in order to circumscribe the ML approach to the genes belonging to the PI3K-AKT-mTOR signaling pathway (WP3844). Upper panel: mean absolute values corresponding tothe magnitude of feature importance. Lower panel: summary plots for SHAP values; for each considered feature, a single patient is represented by one point. Along the x axis the position of a point corresponds to the logarithm of the mortality risk associated with that feature for a specific patients. This value corresponds to the impact that the feature had on the model output for that specific patient. Along the y axis, the different features are disposed according to their importance corresponding to the mean of their absolute SHAP values. Features with the higher importance are disposed on the upper part of the summary plots. Data clusters with SHAP values around zero indicate low impact on the model. SHAP, Shapley Additive exPlanation. B) Circulating barplot recapitulating the contribution in predicting OS of clinical/surgical parameters, TMB values and somatic gene mutations included in the model. **Figure S3.** Impact of TMB on survival in the TCGA high grade glioma series. 1 Kaplan-Meier curves comparing the OS intervals of cases with high TMB (TMB ≥ 1.7, red line) and cases with low TMB (TMB <1.7, blu line). The p-value reported refers to the log-rank test. **Table S1. **Clinical characterization of the GG4 cohort (102 cases). **Table S2.** Categorization of variables in datasets. **Table S3.** Dataset name and composition of the datasets used with the relative reported metrics from xgboost analysis. **Table S4.** Detailed lists of clinical/surgical variables and gene alterations composing datasests employed for the xgboost analysis. 

## Data Availability

Data are available upon request to the corresponding author (Maurizio Polano). The data are not publicly available because they contain information that could compromise the privacy of research participants.

## Abbreviations

AKT: RAC-alpha serine/threonine-protein kinase
BCNU: bis-chloroethylnitrosourea
BRAF: v-Raf murine sarcoma viral oncogene homolog B
CGGA: Chinese Glioma Genome Atlas
c-index: concordance index
CNV: Copy number variations
CW: Carmustine wafers
EGFR: Epidermal growth factor receptor
EOR: Extent of resection
GG4: Glioma grade 4
HR: Hazard ratio
IDH: Isocitrate dehydrogenase
indel: Insertion deletion
MB: Megabase
MGMT: O-6-methylguanine-DNA methyltransferase
ML: Machine learning
MNV: Multi-nucleotide variants
MRI: Magnetic resonance imaging
mTOR: Mammalian target of rapamycin
NGS: Next generation sequencing
NOTCH3: Neurogenic locus notch homolog protein 3
NTRK2: Neurotrophic receptor tyrosine kinase 2
OS: Overall survival
PI3K: PhosphatidylInositol 3-Kinase
POLE: DNA polymerase epsilon, catalytic subunit
PTEN: Phosphatase and tensin homolog
SHAP: Shapley Additive exPlanations
SNV: Single nucleotide variants
TCGA: The Cancer Genome Atlas Program
TMB: Tumor mutational burden
TP53: Tumor protein p53
XGBoost-Surv: eXtreme gradient boosting for survival
WHO: World health organization

## References

[1] Louis DN, Perry A, Reifenberger G, von Deimling A, Figarella-Branger D, Cavenee WK, Ohgaki H, Wiestler OD, Kleihues P, Ellison DW (2016). The 2016 World Health Organization classification of tumors of the central nervous system: a summary. Acta Neuropathol.

[2] Louis DN, Perry A, Wesseling P, Brat DJ, Cree IA, Figarella-Branger D, Hawkins C, Ng HK, Pfister SM, Reifenberger G (2021). The 2021 WHO classification of tumors of the central nervous system: a summary. Neuro Oncol.

[3] Ius T, Sabatino G, Panciani PP, Fontanella MM, Ruda R, Castellano A, Barbagallo GMV, Belotti F, Boccaletti R, Catapano G (2023). Surgical management of Glioma Grade 4: technical update from the neuro-oncology section of the italian society of neurosurgery (SINch(R)): a systematic review. J Neurooncol.

[4] Karschnia P, Young JS, Dono A, Hani L, Sciortino T, Bruno F, Juenger ST, Teske N, Morshed RA, Haddad AF (2022). Prognostic validation of a new classification system for extent of resection in glioblastoma: a report of the RANO resect group. Neuro Oncol.

[5] Ius T, Pignotti F, Della Pepa GM, La Rocca G, Somma T, Isola M, Battistella C, Gaudino S, Polano M, Dal Bo M (2020). A novel comprehensive clinical stratification model to refine prognosis of glioblastoma patients undergoing surgical resection. Cancers (Basel).

[6] Nam JY, de Groot JF (2017). Treatment of glioblastoma. J Oncol Pract.

[7] Stupp R, Hegi ME, Mason WP, van den Bent MJ, Taphoorn MJ, Janzer RC, Ludwin SK, Allgeier A, Fisher B, Belanger K (2009). Effects of radiotherapy with concomitant and adjuvant temozolomide versus radiotherapy alone on survival in glioblastoma in a randomised phase III study: 5-year analysis of the EORTC-NCIC trial. Lancet Oncol.

[8] Stupp R, Mason WP, van den Bent MJ, Weller M, Fisher B, Taphoorn MJ, Belanger K, Brandes AA, Marosi C, Bogdahn U (2005). Radiotherapy plus concomitant and adjuvant temozolomide for glioblastoma. N Engl J Med.

[9] Poon MTC, Sudlow CLM, Figueroa JD, Brennan PM (2020). Longer-term (>/= 2 years) survival in patients with glioblastoma in population-based studies pre- and post-2005: a systematic review and meta-analysis. Sci Rep.

[10] Ius T, Pignotti F, Della Pepa GM, Bagatto D, Isola M, Battistella C, Gaudino S, Pegolo E, Chiesa S, Arcicasa M (2022). Glioblastoma: from volumetric analysis to molecular predictors. J Neurosurg Sci.

[11] Menna G, Manini I, Cesselli D, Skrap M, Olivi A, Ius T, Della Pepa GM (2022). Immunoregulatory effects of glioma-associated stem cells on the glioblastoma peritumoral microenvironment: a differential PD-L1 expression from core to periphery?. Neurosurg Focus.

[12] Di Cintio F, Dal Bo M, Baboci L, De Mattia E, Polano M, Toffoli G (2020). The molecular and microenvironmental landscape of glioblastomas: implications for the novel treatment choices. Front Neurosci.

[13] Pallud J, Audureau E, Noel G, Corns R, Lechapt-Zalcman E, Duntze J, Pavlov V, Guyotat J, Hieu PD, Le Reste PJ (2015). Long-term results of carmustine wafer implantation for newly diagnosed glioblastomas: a controlled propensity-matched analysis of a french multicenter cohort. Neuro Oncol.

[14] Ricciardi L, Manini I, Cesselli D, Trungu S, Piazza A, Mangraviti A, Miscusi M, Raco A, Ius T (2022). Carmustine wafers implantation in patients with newly diagnosed high grade glioma: is it still an option?. Front Neurol.

[15] Brem H, Piantadosi S, Burger PC, Walker M, Selker R, Vick NA, Black K, Sisti M, Brem S, Mohr G (1995). Placebo-controlled trial of safety and efficacy of intraoperative controlled delivery by biodegradable polymers of chemotherapy for recurrent gliomas. The polymer-brain Tumor Treatment Group. Lancet.

[16] Iuchi T, Inoue A, Hirose Y, Morioka M, Horiguchi K, Natsume A, Arakawa Y, Iwasaki K, Fujiki M, Kumabe T, Sakata Y (2022). Long-term effectiveness of gliadel implant for malignant glioma and prognostic factors for survival: 3-year results of a postmarketing surveillance in Japan. Neurooncol Adv.

[17] Wen PY, Weller M, Lee EQ, Alexander BM, Barnholtz-Sloan JS, Barthel FP, Batchelor TT, Bindra RS, Chang SM, Chiocca EA (2020). Glioblastoma in adults: a Society for Neuro-Oncology (SNO) and european Society of Neuro-Oncology (EANO) consensus review on current management and future directions. Neuro Oncol.

[18] Westphal M, Hilt DC, Bortey E, Delavault P, Olivares R, Warnke PC, Whittle IR, Jaaskelainen J, Ram Z (2003). A phase 3 trial of local chemotherapy with biodegradable carmustine (BCNU) wafers (gliadel wafers) in patients with primary malignant glioma. Neuro Oncol.

[19] Ius T, Cesselli D, Isola M, Toniato G, Pauletto G, Sciacca G, Fabbro S, Pegolo E, Rizzato S, Beltrami AP (2018). Combining clinical and molecular data to predict the benefits of carmustine wafers in newly diagnosed high-grade gliomas. Curr Treat Options Neurol.

[20] Brown TJ, Brennan MC, Li M, Church EW, Brandmeir NJ, Rakszawski KL, Patel AS, Rizk EB, Suki D, Sawaya R, Glantz M (2016). Association of the extent of resection with survival in glioblastoma: a systematic review and meta-analysis. JAMA Oncol.

[21] Della Pepa GM, Caccavella VM, Menna G, Ius T, Auricchio AM, Sabatino G, La Rocca G, Chiesa S, Gaudino S, Marchese E, Olivi A (2021). Machine learning-based prediction of early recurrence in glioblastoma patients: a glance towards precision medicine. Neurosurgery.

[22] Weller M, Felsberg J, Hartmann C, Berger H, Steinbach JP, Schramm J, Westphal M, Schackert G, Simon M, Tonn JC (2009). Molecular predictors of progression-free and overall survival in patients with newly diagnosed glioblastoma: a prospective translational study of the German glioma network. J Clin Oncol.

[23] Yan H, Parsons DW, Jin G, McLendon R, Rasheed BA, Yuan W, Kos I, Batinic-Haberle I, Jones S, Riggins GJ (2009). IDH1 and IDH2 mutations in gliomas. N Engl J Med.

[24] Cancer Genome Atlas Research N (2008). Comprehensive genomic characterization defines human glioblastoma genes and core pathways. Nature.

[25] Brennan CW, Verhaak RG, McKenna A, Campos B, Noushmehr H, Salama SR, Zheng S, Chakravarty D, Sanborn JZ, Berman SH (2013). The somatic genomic landscape of glioblastoma. Cell.

[26] Wang J, Cazzato E, Ladewig E, Frattini V, Rosenbloom DI, Zairis S, Abate F, Liu Z, Elliott O, Shin YJ (2016). Clonal evolution of glioblastoma under therapy. Nat Genet.

[27] Wang L, Ge J, Lan Y, Shi Y, Luo Y, Tan Y, Liang M, Deng S, Zhang X, Wang W (2020). Tumor mutational burden is associated with poor outcomes in diffuse glioma. BMC Cancer.

[28] Touat M, Li YY, Boynton AN, Spurr LF, Iorgulescu JB, Bohrson CL, Cortes-Ciriano I, Birzu C, Geduldig JE, Pelton K (2020). Mechanisms and therapeutic implications of hypermutation in gliomas. Nature.

[29] Hodges TR, Ott M, Xiu J, Gatalica Z, Swensen J, Zhou S, Huse JT, de Groot J, Li S, Overwijk WW (2017). Mutational burden, immune checkpoint expression, and mismatch repair in glioma: implications for immune checkpoint immunotherapy. Neuro Oncol.

[30] Sundrani S, Lu J (2021). Computing the hazard ratios associated with explanatory variables using machine learning models of survival data. JCO Clin Cancer Inform.

[31] Moncada-Torres A, van Maaren MC, Hendriks MP, Siesling S, Geleijnse G (2021). Explainable machine learning can outperform Cox regression predictions and provide insights in breast cancer survival. Sci Rep.

[32] Stiglic G, Kocbek P, Fijacko N, Zitnik M, Verbert K, Cilar L (2020). Interpretability of machine learning-based prediction models in healthcare. WIREs Data Min Knowl Discov.

[33] Ellrott K, Bailey MH, Saksena G, Covington KR, Kandoth C, Stewart C, Hess J, Ma S, Chiotti KE, McLellan M (2018). Scalable open science approach for mutation calling of tumor exomes using multiple genomic pipelines. Cell Syst.

[34] Spooner A, Chen E, Sowmya A, Sachdev P, Kochan NA, Trollor J, Brodaty H (2020). A comparison of machine learning methods for survival analysis of high-dimensional clinical data for dementia prediction. Sci Rep.

[35] Pedregosa F, Varoquaux G, Gramfort A, Michel V, Thirion B, Grisel O, Blondel M, Prettenhofer P, Weiss R, Dubourg V (2011). Scikit-learn: machine learning in Python. J Mach Learn Res.

[36] Bergstra J, Yamins D, Cox D. Making a science of model search: hyperparameter optimization in hundreds of dimensions for vision architectures. In Proceedings of the 30th International Conference on Machine Learning (Sanjoy D, David M eds.). Proceedings of Machine Learning Research: PMLR; 2013. 28:115–123.

[37] Jansen T, Geleijnse G, Van Maaren M, Hendriks MP, Ten Teije A, Moncada-Torres A (2020). Machine learning explainability in breast Cancer survival. Stud Health Technol Inform.

[38] Polano M, Chierici M, Dal Bo M, Gentilini D, Di Cintio F, Baboci L, Gibbs DL, Furlanello C, Toffoli G (2019). A pan-cancer approach to predict responsiveness to immune checkpoint inhibitors by machine learning. Cancers (Basel).

[39] Polano M, Fabbiani E, Adreuzzi E, Cintio FD, Bedon L, Gentilini D, Mongiat M, Ius T, Arcicasa M, Skrap M (2021). A new epigenetic model to stratify glioma patients according to their immunosuppressive state. Cells.

[40] Harrell FE, Califf RM, Pryor DB, Lee KL, Rosati RA (1982). Evaluating the yield of medical tests. JAMA.

[41] Edlind MP, Hsieh AC (2014). PI3K-AKT-mTOR signaling in prostate cancer progression and androgen deprivation therapy resistance. Asian J Androl.

[42] Gynecologic oncology (1991). Curr Opin Obstet Gynecol.

[43] Xu J, Xu FP, Liu ZH, Cui Q, Zhang KP, Li Z (2022). The correlation analysis of TERT promoter mutations with IDH1/2 mutations and 1p/19q detected in human gliomas. Med (Baltim).

[44] McNulty SN, Schwetye KE, Ferguson C, Storer CE, Ansstas G, Kim AH, Gutmann DH, Rubin JB, Head RD, Dahiya S (2021). BRAF mutations may identify a clinically distinct subset of glioblastoma. Sci Rep.

[45] Zhang P, Chen X, Zhang L, Cao D, Chen Y, Guo Z, Chen J (2022). POLE2 facilitates the malignant phenotypes of glioblastoma through promoting AURKA-mediated stabilization of FOXM1. Cell Death Dis.

[46] Su LP, Ji M, Liu L, Sang W, Xue J, Wang B, Pu HW, Zhang W (2022). The expression of ASAP3 and NOTCH3 and the clinicopathological characteristics of adult glioma patients. Open Med (Wars).

[47] Bouche V, Aldegheri G, Donofrio CA, Fioravanti A, Roberts-Thomson S, Fox SB, Schettini F, Generali D (2021). BRAF Signaling Inhibition in Glioblastoma: which clinical perspectives?. Front Oncol.

[48] Han F, Hu R, Yang H, Liu J, Sui J, Xiang X, Wang F, Chu L, Song S (2016). PTEN gene mutations correlate to poor prognosis in glioma patients: a meta-analysis. Onco Targets Ther.

[49] Zhang Y, Dube C, Gibert M, Cruickshanks N, Wang B, Coughlan M, Yang Y, Setiady I, Deveau C, Saoud K (2018). The p53 pathway in glioblastoma. Cancers (Basel).

[50] Langhans J, Schneele L, Trenkler N, von Bandemer H, Nonnenmacher L, Karpel-Massler G, Siegelin MD, Zhou S, Halatsch ME, Debatin KM, Westhoff MA (2017). The effects of PI3K-mediated signalling on glioblastoma cell behaviour. Oncogenesis.

[51] Pearson JRD, Regad T (2017). Targeting cellular pathways in glioblastoma multiforme. Signal Transduct Target Ther.

[52] Mallett S, Royston P, Waters R, Dutton S, Altman DG (2010). Reporting performance of prognostic models in cancer: a review. BMC Med.

[53] Villani V, Casini B, Tanzilli A, Lecce M, Rasile F, Telera S, Pace A, Piludu F, Terrenato I, Rollo F (2023). The Glioma-IRE project - molecular profiling in patients with glioma: steps toward an individualized diagnostic and therapeutic approach. J Transl Med.

[54] Merino DM, McShane LM, Fabrizio D, Funari V, Chen SJ, White JR, Wenz P, Baden J, Barrett JC, Chaudhary R (2020). Establishing guidelines to harmonize tumor mutational burden (TMB): in silico assessment of variation in TMB quantification across diagnostic platforms: phase I of the friends of cancer research TMB harmonization project. J Immunother Cancer.

[55] Chalmers ZR, Connelly CF, Fabrizio D, Gay L, Ali SM, Ennis R, Schrock A, Campbell B, Shlien A, Chmielecki J (2017). Analysis of 100,000 human cancer genomes reveals the landscape of tumor mutational burden. Genome Med.

[56] McGrail DJ, Pilie PG, Rashid NU, Voorwerk L, Slagter M, Kok M, Jonasch E, Khasraw M, Heimberger AB, Lim B (2021). High tumor mutation burden fails to predict immune checkpoint blockade response across all cancer types. Ann Oncol.

[57] Zhao J, Chen AX, Gartrell RD, Silverman AM, Aparicio L, Chu T, Bordbar D, Shan D, Samanamud J, Mahajan A (2019). Immune and genomic correlates of response to anti-PD-1 immunotherapy in glioblastoma. Nat Med.

