# Supplementary material for: Machine learning to improve interpretability of clinical, radiological and panel-based genomic data of glioma grade 4 patients undergoing surgical resection
Source: J Transl Med. 2023 Jul 7;21:450. doi: 10.1186/s12967-023-04308-y (PMC10329348; doi:10.1186/s12967-023-04308-y)
Supplement: Supplementary file 1 — Additional file 1: Figure S1. Distribution and classification of the gene alterations found in the 102 sequenced GG4. cases. A) Distribution of the type of alterations (e.g. missense mutations, nonsense mutations, deletions, amplifications, multihits, complex events) and frequencies for the 20 genes most frequently found altered are shown. The number of sequenced cases found altered for each gene is also shown. Figure S2. ML model obtained by considering somatic mutations involving genes belonging to the PI3K-AKT-mTOR signaling pathway (WP3844). A) Feature importance ranked by the "mean absolute magnitude" of the SHAP values of the model obtained for the 71 GG4 cases with available TMB values using a dataset (dataset 4) that included 27 features constituted by 16 genes, 10 clinical and surgical variables and the biomarker TMB. This selection was chosen in order to circumscribe the ML approach to the genes belonging to the PI3K-AKT-mTOR signaling pathway (WP3844). Upper panel: mean absolute values corresponding tothe magnitude of feature importance. Lower panel: summary plots for SHAP values; for each considered feature, a single patient is represented by one point. Along the x axis the position of a point corresponds to the logarithm of the mortality risk associated with that feature for a specific patients. This value corresponds to the impact that the feature had on the model output for that specific patient. Along the y axis, the different features are disposed according to their importance corresponding to the mean of their absolute SHAP values. Features with the higher importance are disposed on the upper part of the summary plots. Data clusters with SHAP values around zero indicate low impact on the model. SHAP, Shapley Additive exPlanation. B) Circulating barplot recapitulating the contribution in predicting OS of clinical/surgical parameters, TMB values and somatic gene mutations included in the model. Figure S3. Impact of TMB on survival in the TCGA high gr [file 12967_2023_4308_MOESM1_ESM.pdf]

Additional information

Machine learning to improve interpretability of clinical, radiological and panel-based genomic data of glioma grade 4 patients undergoing surgical resection.

Description

- **Additional Methods**
- **Additional Figure Legends**

- **Additional Table S1**
- **Additional Table S2**
- **Additional Table S3**
- **Additional Table S4**

- **Additional Figure S1**
- **Additional Figure S2**
- **Additional Figure S3**

Additional Methods

Clinical characterization of the patient cohort

The 102 GG4 patients were enrolled in the case cohort according to the following inclusion criteria: age $\geq$ 18 years; no previous surgery; no preoperative chemo- or radiotherapy; objective evaluation of preoperative tumor volume on MRI images in DICOM format based on post-contrast T1-weighted MRI sequences and T2-weighted MRI sequences; objective estimation of EOR on post-contrast T1-weighted MRI sequences. Cases were excluded from the case cohort if one or more of the following criteria were present: incomplete imaging data, follow-up interval, and multicentric tumors. Clinical, histopathological and molecular data were collected at the time of diagnosis from medical records. Histological examination, immunohistochemistry for Ki67, analysis of the genetic status of *MGMT* promoter and *IDH1/2* genes were performed as previously described [1-5]. Regarding volumetric analysis, all pre and postoperative tumor segmentations were performed manually across all MRI slices using the OsiriX software tool [2, 6].

The achieved EOR in each case was objectively evaluated using preoperative and postoperative MRI images (DICOM format), based on the contrast area of post-contrast T1 MRI sequences, using the below formula: (Pre-operative tumor volume – Post-operative tumor volume)/Pre-operative tumor volume [2, 6].

Regarding post-operative treatment, after surgery, all patients were treated with combinations of concomitant adjuvant radiotherapy and chemotherapy, followed by adjuvant chemotherapy, as recommended by Stupp [7, 8].

Protein–protein interaction (PPI) Network analysis of genes with somatic mutations

The genes name used to create the matrix were mapped by Search Tool for the Retrieval of Interacting Genes (STRING) database (version 10.5) [9], by using Cytoscape (3.8.2) [10]. The PPI network was generated based on the medium confidence score of 0.40. The pathway were selected for importance and for the biological meaning in the context of GG4.

Machine Learning Modeling

EXtreme Gradient Boosting for survival (XGBoost-Surv) XGBoost modeling t was performed in Python (V3.8) using the xgboost and sklearn_surv library partially evaluated using the scikit-learn library [11]. In each of the datasets, string-based categorical variables were converted to numerical values, and each continuous variable was standardized. To perform survival prediction in XGBoost, the survival time in the original dataset, *ti,* was transformed into Ti for each patient (i) according to the censoring information, where *Ci* =1 if patient i was not censored and Ci =-1 otherwise.

$$Ti=Ci\times ti$$

All XGBoost models were trained using the survival Cox objective function. Additionally, for each data set, hyperparameter tuning was performed using the HyperOpt package [12], with 100 evaluation rounds in accordance with parameter ranges that have been previously used in the literature with the exception of capping the min_child_ weight (minimum sum of instance weight), reg_alpha (L1 regularization term on weights), and reg_lambda (L2 regularization term on weights) at 10 rather 100 [13]. As the objective function for hyperparameter tuning, we computed the mean Harrell's concordance index (c-index) using the five-fold cross-validation (CV) approach [14-16]. We optimized

the parameter using HyperOpt and C-index was used as metric. Once the hyperparameters have been identified, the model was subsequently evaluated on the same total data set using five-fold CV as explained above, but with the randomly selected folds being distinct from those used in the hyperparameter tuning process to accurately assess the generalizability of the model [15].

Statistical analyses

All statistical analyses were performed in R environment (v4.2). The primary endpoints was overall survival (OS). OS data were defined as time from surgery and event (death) or end of follow up (censored observation). OS data were available for all the GG4 cases entering the study. For OS analysis, all events were considered as GG4-related, i.e. all deaths were considered as events whatever the cause. OS were estimated using the Kaplan-Meier plots and comparisons between groups were made by means of log-rank test.

References

1. Preusser M, Berghoff AS, Manzl C, Filipits M, Weinhausel A, Pulverer W, Dieckmann K, Widhalm G, Wohrer A, Knosp E, et al: **Clinical Neuropathology practice news 1-2014: pyrosequencing meets clinical and analytical performance criteria for routine testing of MGMT promoter methylation status in glioblastoma.** *Clin Neuropathol* 2014, **33:**6-14.
2. Ius T, Pignotti F, Della Pepa GM, Bagatto D, Isola M, Battistella C, Gaudino S, Pegolo E, Chiesa S, Arcicasa M, et al: **Glioblastoma: from volumetric analysis to molecular predictors.** *J Neurosurg Sci* 2022, **66:**173-186.
3. Ius T, Sabatino G, Panciani PP, Fontanella MM, Ruda R, Castellano A, Barbagallo GMV, Belotti F, Boccaletti R, Catapano G, et al: **Surgical management of Glioma Grade 4: technical update from the neuro-oncology section of the Italian Society of Neurosurgery (SINch(R)): a systematic review.** *J Neurooncol* 2023.
4. Louis DN, Perry A, Reifenberger G, von Deimling A, Figarella-Branger D, Cavenee WK, Ohgaki H, Wiestler OD, Kleihues P, Ellison DW: **The 2016 World Health Organization Classification of Tumors of the Central Nervous System: a summary.** *Acta Neuropathol* 2016, **131:**803-820.
5. Louis DN, Perry A, Wesseling P, Brat DJ, Cree IA, Figarella-Branger D, Hawkins C, Ng HK, Pfister SM, Reifenberger G, et al: **The 2021 WHO Classification of Tumors of the Central Nervous System: a summary.** *Neuro Oncol* 2021, **23:**1231-1251.
6. Ius T, Angelini E, Thiebaut de Schotten M, Mandonnet E, Duffau H: **Evidence for potentials and limitations of brain plasticity using an atlas of functional resectability of WHO grade II gliomas: towards a "minimal common brain".** *Neuroimage* 2011, **56:**992-1000.
7. Stupp R, Hegi ME, Mason WP, van den Bent MJ, Taphoorn MJ, Janzer RC, Ludwin SK, Allgeier A, Fisher B, Belanger K, et al: **Effects of radiotherapy with concomitant and**

adjuvant temozolomide versus radiotherapy alone on survival in glioblastoma in a randomised phase III study: 5-year analysis of the EORTC-NCIC trial. *Lancet Oncol* 2009, **10:**459-466.

8. Stupp R, Mason WP, van den Bent MJ, Weller M, Fisher B, Taphoorn MJ, Belanger K, Brandes AA, Marosi C, Bogdahn U, et al: **Radiotherapy plus concomitant and adjuvant temozolomide for glioblastoma.** *N Engl J Med* 2005, **352:**987-996.
9. Szklarczyk D, Franceschini A, Wyder S, Forslund K, Heller D, Huerta-Cepas J, Simonovic M, Roth A, Santos A, Tsafou KP, et al: **STRING v10: protein-protein interaction networks, integrated over the tree of life.** *Nucleic Acids Res* 2015, **43:**D447-452.
10. Shannon P, Markiel A, Ozier O, Baliga NS, Wang JT, Ramage D, Amin N, Schwikowski B, Ideker T: **Cytoscape: a software environment for integrated models of biomolecular interaction networks.** *Genome Res* 2003, **13:**2498-2504.
11. Pedregosa F, Varoquaux G, Gramfort A, Michel V, Thirion B, Grisel O, Blondel M, Prettenhofer P, Weiss R, Dubourg V, et al: **Scikit-learn: Machine Learning in Python.** *Journal of Machine Learning Research* 2011, **12:**2825-2830.
12. Bergstra J, Yamins D, Cox D: **Making a Science of Model Search: Hyperparameter Optimization in Hundreds of Dimensions for Vision Architectures.** In *Proceedings of the 30th International Conference on Machine Learning* (Sanjoy D, David M eds.), vol. 28. pp. 115--123. Proceedings of Machine Learning Research: PMLR; 2013:115--123.
13. Jansen T, Geleijnse G, Van Maaren M, Hendriks MP, Ten Teije A, Moncada-Torres A: **Machine Learning Explainability in Breast Cancer Survival.** *Stud Health Technol Inform* 2020, **270:**307-311.

14. Polano M, Chierici M, Dal Bo M, Gentilini D, Di Cintio F, Baboci L, Gibbs DL, Furlanello C, Toffoli G: **A Pan-Cancer Approach to Predict Responsiveness to Immune Checkpoint Inhibitors by Machine Learning.** *Cancers (Basel)* 2019, **11**.
15. Moncada-Torres A, van Maaren MC, Hendriks MP, Siesling S, Geleijnse G: **Explainable machine learning can outperform Cox regression predictions and provide insights in breast cancer survival.** *Sci Rep* 2021, **11:**6968.
16. Polano M, Fabbiani E, Adreuzzi E, Cintio FD, Bedon L, Gentilini D, Mongiat M, Ius T, Arcicasa M, Skrap M, et al: **A New Epigenetic Model to Stratify Glioma Patients According to Their Immunosuppressive State.** *Cells* 2021, **10**.

Additional figure legends

Figure S1. Distribution and classification of the gene alterations found in the 102 sequenced GG4 cases. A) Distribution of the type of alterations (e.g. missense mutations, nonsense mutations, deletions, amplifications, multihits, complex events) and frequencies for the 20 genes most frequently found altered are shown. The number of sequenced cases found altered for each gene is also shown.

Figure S2. ML model obtained by considering somatic mutations involving genes belonging to the PI3K-AKT-mTOR signaling pathway (WP3844). A) Feature importance ranked by the "mean absolute magnitude" of the SHAP values of the model obtained for the 71 GG4 cases with available TMB values using a dataset (dataset 4) that included 27 features constituted by 16 genes, 10 clinical and surgical variables and the biomarker TMB. This selection was chosen in order to circumscribe the ML approach to the genes belonging to the PI3K-AKT-mTOR signaling pathway (WP3844). Upper panel: mean absolute values corresponding to the magnitude of feature importance. Lower panel: summary plots for SHAP values; for each considered feature, a single patient is represented by one point. Along the x axis the position of a point corresponds to the logarithm of the mortality risk associated with that feature for a specific patients. This value corresponds to the impact that the feature had on the model output for that specific patient. Along the y axis, the different features are disposed according to their importance corresponding to the mean of their absolute SHAP values. Features with the higher importance are disposed on the upper part of the summary plots. Data clusters with SHAP values around zero indicate low impact on the model. SHAP, Shapley Additive exPlanation. B) Circulating barplot recapitulating the contribution in predicting OS of clinical/surgical parameters, TMB values and somatic gene mutations included in the model.

Figure S3. Impact of TMB on survival in the TCGA high grade glioma series. Kaplan-Meier curves comparing the OS intervals of cases with high TMB (TMB ≥ 1.7, red line) and cases with low TMB (TMB <1.7, blu line). The p-value reported refers to the log-rank test.

Table S1. Clinical characterization of the GG4 cohort (102 cases).

**Characteristic**	**number of cases (102)**
CW treatment, n (%)	
0; not-treated	63 (62%)
1; treated	39 (38%)
Age, Median (IQR)	60 (52 – 69)
EOR, Median (IQR)	98% (95% – 100%)
Localization, n (%)	
0; precentral	41 (40%)
1;postcentral	21 (21%)
2; temporoinsular	40 (39%)
Ki67, Median (IQR)	40 (24 – 60)
IDH mutated status, n (%)	
0; unmutated	94 (92.2%)
1; mutated	8 (7.8%)
side, n (%)	
0; left	54 (53%)
1; right	48 (47%)
Pre-operative volume, Median (IQR)	32 (19 – 46)
Residual volume, Median (IQR)	0.14 (0.00 – 1.65)

Extent of resection_2categories, n (%)	
0; ≤99%	52 (51%)
1; ≥100%	50 (49%)
MGMT status, n (%)	
0; unmethylated	40 (39%)
1; methylated	62 (61%)

Abbreviations: IQR, interquartile range; CW, carmustine wafer; EOR, extent of resection.

Table S2. Categorization of variables in datasets.

Category	Category name	Variable name
**Radiological variables**	Clinical	Extent of Resection (EOR), residual volume, preoperative volume.
**Clinical variables**	Clinical	Age at diagnosis, side, localization, *IDH* mutational status, *MGMT* promoter methylation status, gender, carmustine wafer (CW) treatment.
**Mutation Status**	Molecular	Somatic mutations in genes present in the TSO500 Illumina.
**Amplification**	Molecular	Amplification reported in genes of TSO500 Illumina.
**Tumor Mutational Burden**	TMB	Tumor Mutational Burden.

Table S3.

Dataset name and composition of the datasets used with the relative reported metrics from xgboost analysis.

**Dataset**	**Number Cases**	**Dataset Composition**	**Count**	**C-index test**	**C-index CV (CI)**
**Dataset 1**	102	417 gene mutations, 45 gene amplifications, 12 clinical and radiological variables	474	0.540	0.696 (0.655-0.754)
**Dataset 2**	102	95 gene mutations, 12 clinical and radiological variables	107	0.682	0.674 (0.610-0.734)
**Dataset 3**	71	95 gene mutations, 12 clinical and radiological variables, TMB	108	0.625	0.646 (0.619-0.674)
**Dataset 4**	71	16 gene mutations, 10 clinical and radiological variables, TMB	27	0.670	0.657 (0.577-0.746)

Abbreviations: CI, confidence interval; TMB, tumor mutational burden.

Table S4. Detailed lists of clinical/surgical variables and gene alterations composing datasests employed for the xgboost analysis.

Dataset 1 (102 GG4 cases)	Dataset 2 (102 GG4 cases)	Dataset 3 (71 GG4 cases)	Dataset 4 (71 GG4 cases)
ABRAXAS1	ALK	ALK	ATM
ACVR1	ANKRD11	ANKRD11	BRAF
ACVR1B	ARID1B	ARID1B	BRCA2
ADGRA2	ARID2	ARID2	CBL
AKT1	ATM	ATM	EGFR
AKT2	ATR	ATR	MET
ALK	ATRX	ATRX	MSH6
ALOX12B	AXIN2	AXIN2	NF1
AMER1	AXL	AXL	PIK3C2G
ANKRD11	BCL6	BCL6	PIK3CA
ANKRD26	BLM	BLM	PIK3R1
APC	BRAF	BRAF	PLCG2
AR	BRCA2	BRCA2	PTEN
ARAF	BRD4	BRD4	RB1
ARFRP1	BRIP1	BRIP1	TP53
ARID1A	CARD11	CARD11	TSC2
ARID1B	CBL	CBL	CW treatment
ARID2	CDK12	CDK12	IDH mutated status
ARID5B	CREBBP	CREBBP	MGMT promoter methylated status
ASXL1	CSF1R	CSF1R	Gender_female
ASXL2	CUX1	CUX1	Age
ATM	DICER1	DICER1	Localization
ATR	DNMT3A	DNMT3A	side
ATRX	DNMT3B	DNMT3B	Ki67
AURKA	EGFR	EGFR	Pre-operative volume
AURKB	EPHA3	EPHA3	Residual volume
AXIN1	EPHA5	EPHA5	TMB
AXIN2	EPHB1	EPHB1	
AXL	ERCC2	ERCC2	
B2M	ERCC3	ERCC3	
BAP1	ERCC4	ERCC4	
BARD1	FANCA	FANCA	
BBC3	FANCD2	FANCD2	
BCL10	FAT1	FAT1	
BCL2L11	FGFR4	FGFR4	
BCL2L2	GABRA6	GABRA6	
BCL6	GEN1	GEN1	
BCOR	GNAS	GNAS	
BCORL1	GRIN2A	GRIN2A	
BCR	IDH1	IDH1	
BIRC3	INSR	INSR	
BLM	JAK2	JAK2	
BMPR1A	KDM5A	KDM5A	
BRAF	LATS1	LATS1	
BRCA1	LRP1B	LRP1B	
BRCA2	LZTR1	LZTR1	
BRD4	MAP3K1	MAP3K1	
BRIP1	MDC1	MDC1	
CALR	MED12	MED12	
CARD11	MET	MET	
CASP8	MGA	MGA	
CBL	MSH3	MSH3	
CCND3	MSH6	MSH6	
CCNE1	MST1	MST1	
CD274	MST1R	MST1R	
CD276	MTOR	MTOR	
CD74	NCOR1	NCOR1	
CD79A	NF1	NF1	
CDH1	NFE2L2	NFE2L2	
CDK12	NOTCH1	NOTCH1	
CDK4	NOTCH2	NOTCH2	
CDK6	NOTCH3	NOTCH3	
CDKN1A	NOTCH4	NOTCH4	
CDKN1B	NTRK1	NTRK1	
CDKN2A	NUP93	NUP93	
CDKN2C	NUTM1	NUTM1	
CEBPA	PBRM1	PBRM1	
CHD2	PIK3C2G	PIK3C2G	

CHD4	PIK3CA	PIK3CA
CHEK1	PIK3R1	PIK3R1
CHEK2	PLCG2	PLCG2
CIC	POLD1	POLD1
COP1	POLE	POLE
CREBBP	PTCH1	PTCH1
CRLF2	PTEN	PTEN
CSF1R	PTPN11	PTPN11
CSF3R	PTPRT	PTPRT
CTLA4	RANBP2	RANBP2
CTNNA1	RB1	RB1
CUL3	ROS1	ROS1
CUX1	RPTOR	RPTOR
CXCR4	SDHA	SDHA
CYLD	SETBP1	SETBP1
DAXX	SETD2	SETD2
DDR2	SLIT2	SLIT2
DHX15	SLX4	SLX4
DICER1	SPEN	SPEN
DIS3	SPTA1	SPTA1
DNAJB1	TCF3	TCF3
DNMT1	TET1	TET1
DNMT3A	TET2	TET2
DNMT3B	TP53	TP53
DOT1L	TSC2	TSC2
E2F3	ZFHX3	ZFHX3
EGFL7	ZNF217	ZNF217
EGFR	CW treatment	CW treatment
EIF4A2	Extent Of Resection _ 2 categories	Extent Of Resection _ 2 categories
EIF4E	IDH mutated status	IDH mutated status
EML4	MGMT promoter methylated status	MGMT promoter methylated status
EMSY	Gender_female	Gender_female
EP300	Age	Age
EPCAM	Localization	Localization
EPHA3	side	side
EPHA5	Ki67	Ki67
EPHA7	EOR	EOR
EPHB1	Preoperative volume	Pre-operative volume
ERBB2	Residual volume	Residual volume
ERBB3		TMB
ERBB4		
ERCC1		
ERCC2		
ERCC3		
ERCC4		
ERCC5		
ERG		
ERRFI1		
ESR1		
ETS1		
ETV1		
ETV4		
ETV5		
ETV6		
EWSR1		
EZH2		
FAM46C		
FANCA		
FANCC		
FANCD2		
FANCE		
FANCF		
FANCG		
FANCI		
FANCL		
FAS		
FAT1		
FBXW7		
FGF1		
FGF10		
FGF23		
FGF3		

FGF4
FGF5
FGF6
FGF8
FGFR1
FGFR2
FGFR3
FGFR4
FLCN
FLI1
FLT1
FLT3
FLT4
FOXA1
FOXP1
FRS2
FYN
GABRA6
GATA1
GATA2
GATA3
GATA4
GATA6
GEN1
GLI1
GNAS
GREM1
GRIN2A
GRM3
H3F3A
H3F3C
HGF
HIST1H1C
HIST1H2BD
HIST1H3B
HIST1H3J
HIST3H3
HNF1A
HOXB13
HRAS
HSD3B1
HSP90AA1
ICOSLG
ID3
IDH1
IDH2
IFNGR1
IGF1R
IGF2
IKBKE
IKZF1
IL7R
INHA
INHBA
INPP4A
INPP4B
INSR
IRF2
IRS1
IRS2
JAK1
JAK2
JAK3
JUN
KAT6A
KDM5A
KDM5C
KDM6A
KDR
KEAP1
KEL
KIF5B

KIT
KLF4
KLHL6
KMT2A
KRAS
LATS1
LATS2
LRP1B
LZTR1
MAGI2
MAP2K1
MAP2K2
MAP3K1
MAP3K13
MAP3K14
MAP3K4
MAPK3
MCL1
MDC1
MDM2
MDM4
MED12
MEN1
MET
MGA
MITF
MLH1
MPL
MRE11
MSH2
MSH3
MSH6
MST1
MST1R
MTOR
MUTYH
MYB
MYC
MYCL
MYD88
MYOD1
NAB2
NBN
NCOA3
NCOR1
NEGR1
NF1
NF2
NFE2L2
NKX2-1
NKX3-1
NOTCH1
NOTCH2
NOTCH3
NOTCH4
NRG1
NSD1
NTRK1
NTRK3
NUP93
NUTM1
PAK1
PAK5
PALB2
PARP1
PAX3
PAX5
PAX7
PAX8
PBRM1
PDCD1
PDCD1LG2

PDGFRA
PDGFRB
PDPK1
PGR
PHF6
PIK3C2B
PIK3C2G
PIK3C3
PIK3CA
PIK3CB
PIK3CD
PIK3CG
PIK3R1
PIK3R2
PIK3R3
PIM1
PLCG2
PLK2
PMAIP1
PMS1
PMS2
PNRC1
POLD1
POLE
PPARG
PPM1D
PPP2R1A
PPP2R2A
PPP6C
PRDM1
PREX2
PRKAR1A
PRKDC
PRKN
PTCH1
PTEN
PTPN11
PTPRD
PTPRS
PTPRT
RAD50
RAD51
RAD51B
RAD51C
RAD51D
RAD52
RAD54L
RAF1
RANBP2
RASA1
RB1
RBM10
RECQL4
REL
RET
RICTOR
RIT1
RNF43
ROS1
RPS6KB1
RPS6KB2
RPTOR
RUNX1
RUNX1T1
SDHA
SDHB
SDHC
SDHD
SETBP1
SETD2
SF3B1
SH2B3

SHQ1
SLIT2
SLX4
SMAD3
SMAD4
SMARCA4
SMARCD1
SMC3
SMO
SNCAIP
SOCS1
SOX17
SOX2
SOX9
SPEN
SPOP
SPTA1
SRC
STAG1
STAG2
STAT5A
STAT5B
STK11
STK40
SUZ12
SYK
TAF1
TBX3
TCF3
TCF7L2
TET1
TET2
TFRC
TGFBR2
TMEM127
TMPRSS2
TNFAIP3
TNFRSF14
TOP2A
TP53
TP63
TRAF2
TRAF7
TSC1
TSC2
TSHR
VEGFA
VHL
VTCN1
WISP3
WT1
XIAP
XRCC2
YES1
ZBTB2
ZFHX3
ZNF217
ZNF703
ZRSR2
ampl:::AKT2
ampl:::ALK
ampl:::AR
ampl:::BRAF
ampl:::BRCA1
ampl:::BRCA2
ampl:::CCND1
ampl:::CCND3
ampl:::CCNE1
ampl:::CDK4
ampl:::CDK6
ampl:::CHEK2
ampl:::EGFR

ampl:::ERBB3
ampl:::ERCC1
ampl:::ERCC2
ampl:::FGF1
ampl:::FGF10
ampl:::FGF14
ampl:::FGF19
ampl:::FGF2
ampl:::FGF23
ampl:::FGF3
ampl:::FGF4
ampl:::FGF6
ampl:::FGF7
ampl:::FGFR1
ampl:::KIT
ampl:::KRAS
ampl:::LAMP1
ampl:::MDM2
ampl:::MDM4
ampl:::MET
ampl:::MYC
ampl:::MYCL
ampl:::MYCN
ampl:::NRAS
ampl:::PDGFRA
ampl:::PDGFRB
ampl:::PIK3CA
ampl:::PIK3CB
ampl:::RAF1
ampl:::RET
ampl:::RICTOR
ampl:::RPS6KB1
CW treatment
Extent Of Resection _ 2 categories
IDH mutated status
MGMT promoter methylated status
Gender_female
Age
Localization
side
Ki67
EOR
Preoperative volume
Residual volume

Abbreviations: CW, carmustine wafer; EOR, extent of resection; TMB, tumor mutational burden.

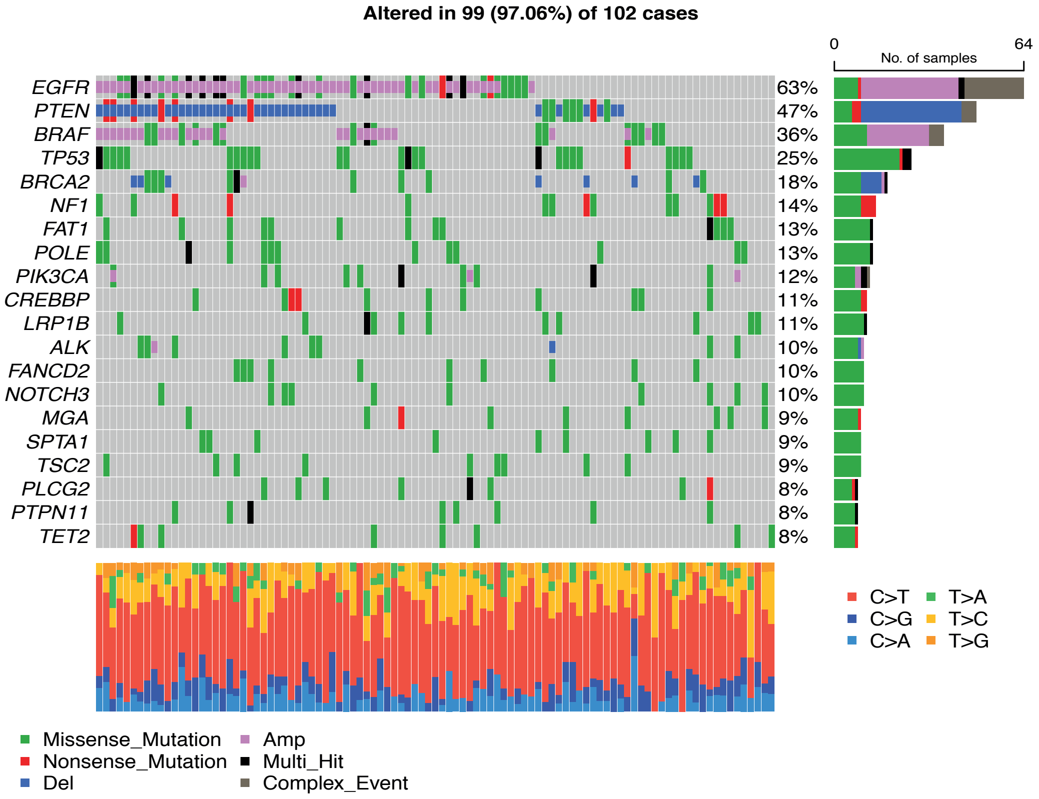
Altered in 99 (97.06%) of 102 cases
0
No. of samples
64
EGFR 63%
PTEN 47%
BRAF 36%
TP53 25%
BRCA2 18%
NF1 14%
FAT1 13%
POLE 13%
PIK3CA 12%
CREBBP 11%
LRP1B 11%
ALK 10%
FANCD2 10%
NOTCH3 10%
MGA 9%
SPTA1 9%
TSC2 9%
PLCG2 8%
PTPN11 8%
TET2 8%
C>T
T>A
C>G
T>C
C>A
T>G
Missense_Mutation
Nonsense_Mutation
Del
Amp
Multi_Hit
Complex_Event

FIGURE S1

A

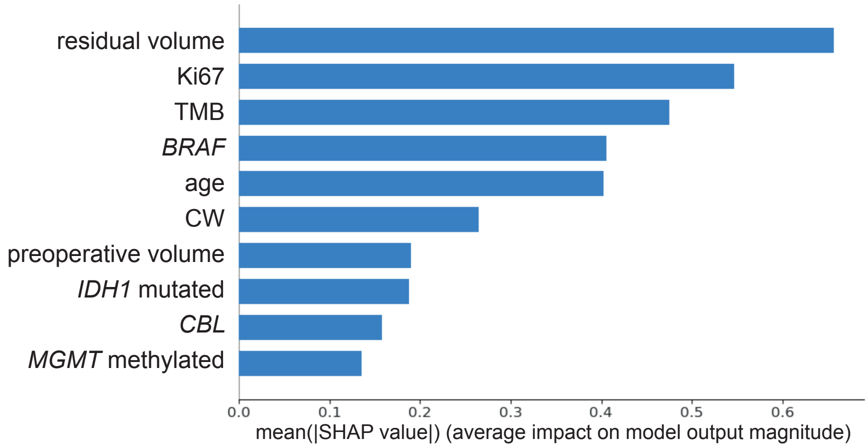
residual volume
Ki67
TMB
BRAF
age
CW
preoperative volume
IDH1 mutated
CBL
MGMT methylated
0.0
0.1
0.2
0.3
0.4
0.5
0.6
mean(|SHAP value|) (average impact on model output magnitude)

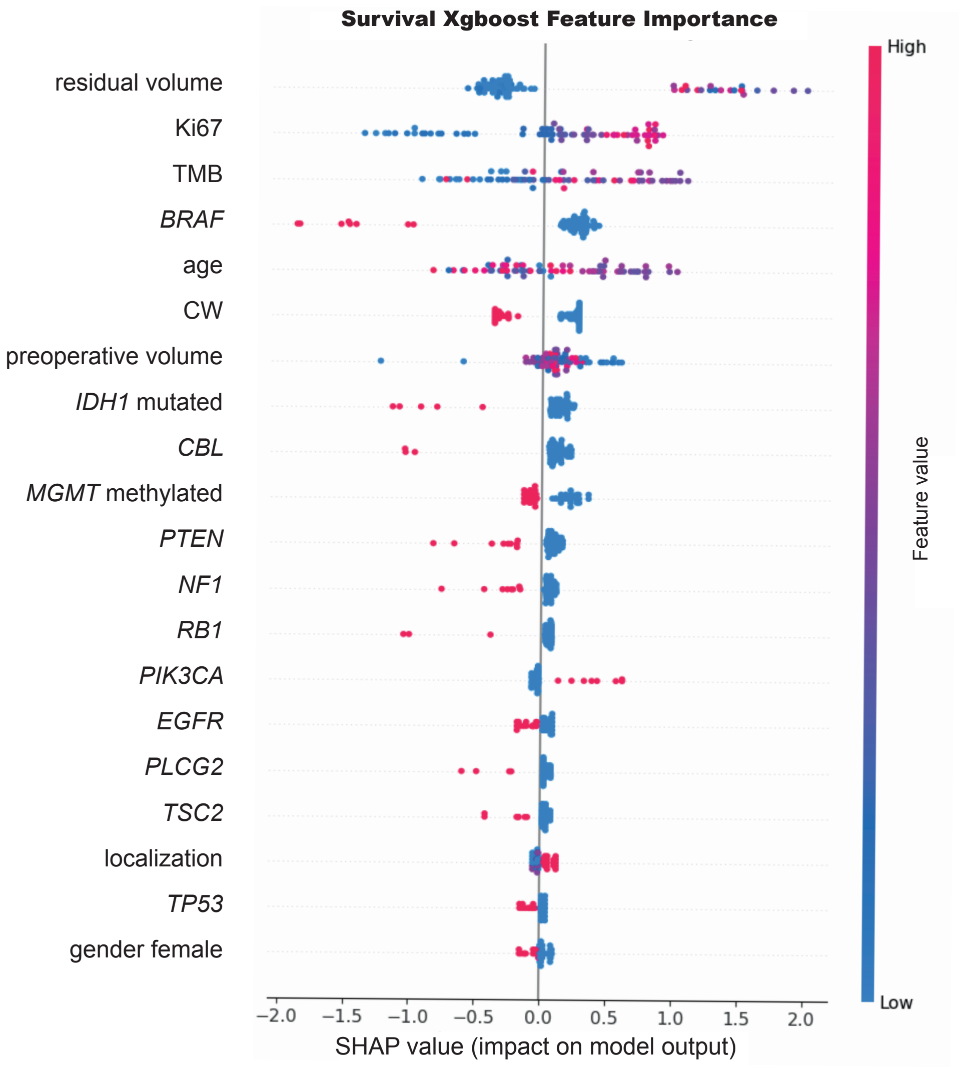
Survival Xgboost Feature Importance
residual volume
Ki67
TMB
BRAF
age
CW
preoperative volume
IDH1 mutated
CBL
MGMT methylated
PTEN
NF1
RB1
PIK3CA
EGFR
PLCG2
TSC2
localization
TP53
gender female
−2.0
−1.5
−1.0
−0.5
0.0
0.5
1.0
1.5
2.0
SHAP value (impact on model output)
High
Low
Feature value

B

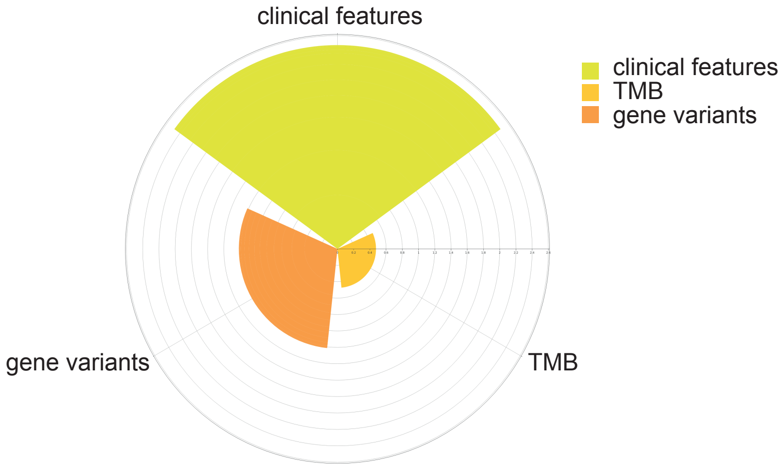
clinical features
gene variants
TMB
clinical features
TMB
gene variants

FIGURE S2

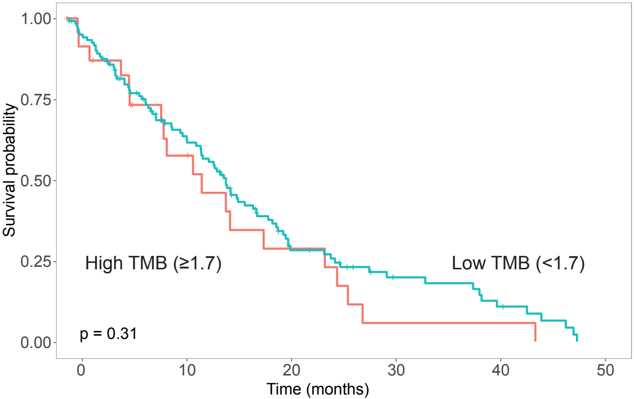

1.00
0.75
0.50
0.25
0.00
Survival probability
High TMB (≥1.7)
Low TMB (<1.7)
p = 0.31
0
10
20
30
40
50
Time (months)

FIGURE S3
